# Identifying errors in dust models from data assimilation

**DOI:** 10.1002/2016GL070621

**Published:** 2016-09-03

**Authors:** R. J. Pope, J. H. Marsham, P. Knippertz, M. E. Brooks, A. J. Roberts

**Affiliations:** ^1^ Institute for Atmospheric and Climate ScienceUniversity of LeedsLeedsUK; ^2^ National Centre for Earth ObservationUniversity of LeedsLeedsUK; ^3^ National Centre for Atmospheric ScienceUniversity of LeedsLeedsUK; ^4^ Institute of Meteorology and Climate ResearchKarlsruhe Institute of TechnologyKarlsruheGermany; ^5^ Met OfficeExeterUK

**Keywords:** aerosol optical depth, dust forecasts, haboobs, data assimilation increments

## Abstract

Airborne mineral dust is an important component of the Earth system and is increasingly predicted prognostically in weather and climate models. The recent development of data assimilation for remotely sensed aerosol optical depths (AODs) into models offers a new opportunity to better understand the characteristics and sources of model error. Here we examine assimilation increments from Moderate Resolution Imaging Spectroradiometer AODs over northern Africa in the Met Office global forecast model. The model underpredicts (overpredicts) dust in light (strong) winds, consistent with (submesoscale) mesoscale processes lifting dust in reality but being missed by the model. Dust is overpredicted in the Sahara and underpredicted in the Sahel. Using observations of lighting and rain, we show that haboobs (cold pool outflows from moist convection) are an important dust source in reality but are badly handled by the model's convection scheme. The approach shows promise to serve as a useful framework for future model development.

## Key Points

- Increments in AOD from data assimilation reveal sources of dust model error
- Dust underpredicted/overpredicted in light/strong winds and missed from convection
- Dust overpredicted/underpredicted in Sahara/Sahel

## 1 Introduction

Mineral dust is an important component of the Earth system [*Knippertz and Todd*, 2012], scattering and absorbing both solar and infrared radiation and affecting cloud microphysics. Dust is increasingly being predicted prognostically within numerical weather prediction (NWP) models because of its impacts on atmospheric circulation [*Mulchay et al.*, 2014; *Solomos et al.*, 2011; *Milton et al.*, 2008] and the boundary layer (BL) [e.g., *Rémy et al.*, 2015]. In addition, many applications such as visibility (e.g., aviation and military activities), air quality, solar energy production, and health (respiratory diseases and meningitis outbreaks) [*Molesworth et al.*, 2002; *Martiny and Chiapello*, 2013] use dust forecasts [*Knippertz and Stuut*, 2014]. In recent years dust models have started to assimilate aerosol optical depth (AOD) from satellite measurements [e.g., *Liu et al.*, 2011; *Niu et al.*, 2008]. The resulting data assimilation increments (DAIs), i.e., the amount of change made to the first guess by the model through the incorporation of observations, present a new opportunity to assess the characteristics and sources of error in dust models, as shown in this study.

Dust emission results from wind stress over dry surfaces with adequate soil characteristics. Associated meteorological phenomena include haboobs (dusty cold pool outflows from deep convective precipitating events initiated in the Sahel and Sahara during the monsoon season) [*Roberts and Knippertz*, 2014; *Miller et al.*, 2008], small‐scale dry convective processes such as dust devils and dusty plumes (DD) [*Balme and Greeley*, 2006; *Marsham et al.*, 2008b], and the breakdown of the nocturnal low‐level jet (NLLJ) [*Fiedler et al.*, 2013; *Parker et al.*, 2005]. Haboobs and NLLJs appear to account for similar amounts of dust emission over West Africa in summer (approximately 40 to 50%) [*Heinold et al.*, 2013; *Marsham et al.*, 2013; *Allen et al.*, 2013], while DDs account for 3.4% (uncertainty range 0.9–31%) of the global dust emission [*Jemmett‐Smith et al.*, 2015]. During the dry season the synoptic‐scale northeasterly Harmattan winds, and the breakdown of the NLLJ within them, cause significant dust uplift especially from the Bodélé Depression [*Washington et al.*, 2006]. Seasonally changing land surface properties (e.g., soil moisture and vegetation) also control dust emission, especially in the Sahel [*Cowie et al.*, 2013; *Pierre et al.*, 2015].

Model uncertainties in the dust cycle are large. For a multimodel ensemble, *Huneeus et al.* [2011] estimated annual global dust emission and deposition to be 200–4000 Tg/yr and 700–4000 Tg/yr, respectively. Observational studies [e.g., *Rajot*, 2001] have also found large uncertainties in quantifying dust emission and deposition, and recently, attempts have been made to use data assimilation to better constrain dust emissions [*Escribano et al.*, 2016]. The differences in modeled emissions are attributed to uncertainty in dust parameterizations (e.g., particle entrainment and suspension) [*Bagnold*, 1941], the land surface [*Kok et al.*, 2014], and representation of model meteorology, often a dominating factor [e.g., *Menut*, 2008; *Heinold et al.*, 2013]. The relationship between wind speed and dust emission is highly nonlinear and usually represented as the third power of the difference between surface wind speed and a fixed threshold friction velocity [e.g., *Marticorena and Bergametti*, 1995], making it sensitive to the tail of the wind speed distribution [*Cowie et al.*, 2015]. Global models are known to underestimate the dust‐lifting power of wind [*Marsham et al.*, 2011; *Knippertz and Todd*, 2012]. *Fiedler et al.* [2013] and *Largeron et al.* [2015] found that meteorological analyses underestimate the stronger NLLJs seen in radiosonde observations. In operational dust models parameterized convection leads to missing haboobs [*Marsham et al.*, 2011] and DDs are too small for all NWP models.

From 30 April 2013 the UK Met Office started assimilating AOD at 550 nm, from the Moderate Resolution Imaging Spectroradiometer (MODIS) instrument aboard NASAs EOS‐AQUA satellite, into its global NWP model. A full year of simulation is used to investigate model error, specifically concentrating on high‐ and low‐wind regimes and haboobs over northern Africa. Section 2 gives a description of the model, assimilation methodology, and the observational data. In section 3 the model AOD and DAI AOD are systematically investigated. Conclusions are provided in section 4.

## 2 Model and Data

We use a global configuration of the Met Office Unified Model (MetUM). The MetUM has a nonhydrostatic, fully compressible, deep‐atmosphere dynamical core, solved with a semi‐implicit semi‐Lagrangian time step on a regular longitudinal‐latitudinal grid [*Davies et al.*, 2005]. The scientific documentation of the MetUM configuration during this period is the Global Atmosphere 3.1 described in detail in *Walters et al.* [2011]. Of particular relevance to this study are the parameterizations of turbulent mixing and convection. The former is parameterized using the first‐order scheme of *Lock et al.* [2000] with additional nonlocal fluxes in convective boundary layers and an explicit parameterization of entrainment mixing across the boundary layer top. This produces a surface friction velocity (U*), which is the meteorological input into the dust emission scheme, representing the effect of surface roughness and turbulent mixing on surface exchange. Convection is parameterized using the mass flux convection scheme based on *Gregory and Rowntree* [1990], extended to include downdrafts and convective momentum transport. The surface fluxes are enhanced by the downdraft mass flux using the parameterization of *Redelsperger et al.* [2000], but the impact of this is small. Over West Africa these parameterizations are known to produce rainfall too early in the day [*Birch et al.*, 2014b], change the sign in the soil moisture rainfall coupling [*Taylor et al.*, 2013], and are unable to produce convectively generated cold pools important for convective organization and dust uplift [*Marsham et al.*, 2011; *Pantillon et al.*, 2015].

The model was run with a horizontal grid spacing of 0.35° longitude by 0.23° latitude (approximately 25 km in midlatitudes and 40 km at the equator), with 70 levels between the surface and 80 km, with terrain‐following coordinates close to the surface and parallel levels at height. Meteorological fields are initialized from initial conditions from 3 h into the previous forecast (*T* + 3), which are modified using a Hybrid Ensemble 4D‐Var data assimilation (DA) process [*Clayton et al.*, 2013]. The DA process produces a set of increments to the main model prognostic fields in order to constrain the current forecast at *T* + 0 to produce the best fit against all of the observations in a 3 h window either side of *T* + 0. This *T* + 0 state is known as the analysis. Due to its computational expense, the DA is run at a lower resolution than the forecast and during the study period had a grid spacing of 0.833° longitude by 0.555° latitude (approximately 60 km in midlatitudes and 90 km at the equator).

The dust component in the MetUM is from *Woodward* [2001] and *Woodward* [2011], based on *Marticorena and Bergametti* [1995], and was developed initially for climate applications (e.g., HadGEM2) [*Martin et al.*, 2011]. Emission is a cubic function of the exceedance of the friction velocity over bare soil (U*) over threshold value (*U*_t_*). U* is determined from the model wind field and boundary layer structure, with an initial horizontal particle flux (i.e., saltation) in nine size bins with diameters from 0.0632 μm to 2000 μm and a subsequent vertical flux into the atmosphere. Only two dust bins are transported, with diameters of 0.2–4 μm and 4–40 μm. The prescribed size distributions in each bin were calculated using an equally weighted mixture of the transported dust and dust storm size distributions from *d'Almeida* [1987], which was found to produce the best fit against aircraft observations.

The land surface specification is of particular relevance to the dust emission. The model soil moisture is initialized by assimilating soil wetness observations from advanced scatterometer on the forecast model grid [*Dharssi et al.*, 2011]. The soil properties are held constant and are derived from *FAO et al.* [2009] data, while the bare soil roughness length is a constant global value of 1 mm. The land fractions for the nonvegetated surface types and the different JULES plant‐functional types are derived from the International Global Biosphere Programme [*Loveland and Belward*, 1997], as is an estimate of the plant canopy heights and therefore the vegetation roughness lengths. The leaf area index (LAI) is updated at the start of every forecast from a monthly climatology derived from MODIS observations [*Knyazikhin et al.*, 1999]. Therefore, LAI and surface roughness evolve through the seasonal cycle while bare soil fraction does not.

While the Woodward [2001] dust model has a sound physical basis, its final performance is dependent on three tuning parameters: one scales the model soil moisture to account for the difference between a model soil level 10 cm deep and the thin surface layer which is relevant for dust emission. The second scales the input U* to account for changes in the wind distribution at different model resolutions or surface roughness specifications, and the final tuning parameter globally scales the dust emission. The representation of wet and dry deposition processes is as described in Woodward [2001], with coefficients modified for the two transported size bins.

Model initial conditions were available at *T* + 3*Z* (03, 09, 15, and 21 UTC) and *T* + 6*Z* (00, 06, 12, and 18 UTC), respectively, and DAIs at 00, 06, 12, and 18 UTC. The DAIs are based on the assimilation of MODIS AOD, which include collection 5.1 (C5) standard MODIS dark target retrievals [*Kaufman et al.*, 1997; *Tanré et al.*, 1997; *Remer et al.*, 2005] over land and sea and Deep Blue retrievals [*Hsu et al.*, 2004, 2006; *Ginoux et al.*, 2010] over desert and arid land surface. In order to remove nondust aerosol AOD observations, the MODIS dark target observations are filtered using the aerosol‐type indicators within the MODIS data files, and the Deep Blue observations are currently assumed to be of dust as these are over desert/arid land surfaces. AQUA has an approximate overpass time of 1330 LT, such that DAIs with useful information are those at 12 UTC. We use these DAIs to assess the dust forecast errors over 1 year, for Africa north of the equator, between 1 May 2013 and 30 April 2014. We focus on the monsoon (May–September) and nonmonsoon (October–April) seasons. The assimilation calculates DAIs on all model levels, based on the AOD column measurement. Therefore, column integrals of the DAIs are presented for consistency with the observations. Assimilation of MODIS AOD changes the model's aerosol fields, altering the radiation, which has a small indirect impact on the meteorology.

**Figure 1:**
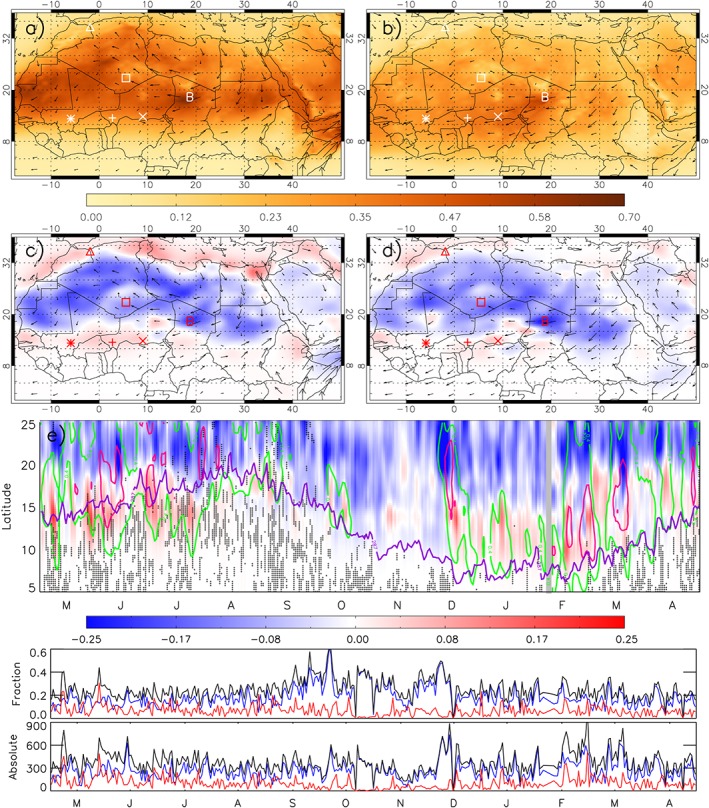
Global model mean dust aerosol optical depth (AOD) at 550 nm at 12 UTC and the data assimilation increment (DAI) AOD, with 850 hPa winds overplotted. (a and c) The monsoon season (May–September 2013) and (b and d) the nonmonsoon season (October 2013 to April 2014). (e) The seasonal cycle (Hovmöller) of the 3 day running mean DAI AOD, averaged longitudinally between 5°W to 15°E. Black crosses = lightning. Green (0.4) and pink (0.6) contours show model AOD. Purple line shows the monsoon front (ITD, 2 m dew point = 14°C). Below are the daily absolute fractional (DAI/model AOD) and total DAIs over the subdomain (5°W–15°E, 5–25°N). Red = positive, blue = negative, and black = total absolute DAI.

For independent model evaluation, AERONET (Aerosol Robtic Network) data are used as unassimilated measures of AOD [*Dubovik et al.*, 2000]. MODIS AODs were retrieved at 550 nm, but limited AERONET data were available at this wavelength. Therefore, the Angstrom exponent [*Seinfeld and Pandis*, 2006] was used to interpolate AERONET AODs to 550 nm. At each site (Figure 1), the closest AERONET observation within 1 h to 12 UTC was sampled to compare with the model AOD. The assimilation of MODIS AODs improves the correlation between the model and AERONET data at all locations, reducing mean bias everywhere except Tamanrasset; see supporting information. To investigate haboobs, we use two independent proxies (lightning and rainfall) from the Lightning Imaging Sensor (LIS) [*Cecil et al.*, 2014] and the Tropical Rainfall Measuring Mission (TRMM).

## 3 Results

### 3.1 Mean Conditions

Figure 1 shows that there are widespread high AODs during the monsoon season (Figure 1a), with maxima between 0.5 and 0.7 over the Bodélé Depression (marked with B) and western Mauritania. Under nonmonsoon conditions (Figure 1b), the dust loading is generally lower, with Saharan AODs between 0.2 and 0.35, peaking again over the Bodélé Depression at 0.4. The 850 hPa winds show Harmattan wind‐driven dust transport into the Sahel and out to the tropical Atlantic.

In both monsoon and nonmonsoon seasons the DAIs show that the model overestimates AOD in the Sahara by around 0.05–0.25 (blue in Figures 1c and 1d). This is on the order of 30% and therefore a significant error (middle panel of Figure 1e). During the monsoon season dust is underestimated over the Sahel, and over the North African coastline and Mediterranean Sea (Figure 1c), and this is seen to a lesser extent in the winter season (Figure 1d). The error over the sea is from transport or deposition since there is no local source. From Figure 1 alone it is not possible to tell whether errors over the Sahel are from emission, transport, or deposition.

Figure 1e shows the AOD maxima (green and purple contours) following the seasonal progression of the monsoon front (the Intertropical Discontinuity, ITD; purple line) as seen by *Engelstaedter and Washington* [2007]. The dust minimum occurs in September–November followed by a maximum in December–March, at 10–15°N, linked to dust emission from the Harmattan winds and the Bodélé Depression NLLJ [*Slingo et al.*, 2006; *Washington et al.*, 2006]. The subsequent JJA summertime maximum is linked with emission from NLLJ and haboobs [*Marsham et al.*, 2008a], and early in this period, dust is uplifted from the dry Sahel land surface [*Cowie et al.*, 2013] and high AODs extend south of the ITD.

Between 20 and 25°N the model overestimates dust in all seasons as shown by the negative DAIs (see also Figures 1c and 1d). In May–August, the northward progression of positive DAIs on either side of the ITD is potentially linked to missing haboob dust emission, caused by the parameterization of convection, which cannot produce the spatial separation of updraft and downdraft in sheared environments that ultimately lead to larger cold pools, organization, and longer lifetimes of convective systems. This is supported by the associated lightning (crosses in Figure 1e) and is discussed in detail in section 3.3. In this time of year and region, where soil moisture and vegetation cover change rapidly, errors in the land surface submodel could be important, too [e.g., *Cowie et al.*, 2013]. In September‐October‐November (SON), there is hardly any significant underprediction in the considered region at any latitude, but from December to April underprediction occurs again to the north of the ITD, where we expect the influence of Harmattan winds and NLLJs. Land surface errors may play a role here, as this marks the period of vegetation dieback in the Sahel.

The model errors are relatively stable throughout the year with marked individual spikes, both for absolute values and for errors considered as a fraction of total AOD. As the model typically overestimates dust, the negative DAIs make up a larger proportion of the model AOD, especially in SON where the AOD is lower and errors are dominated by dust overestimation. The positive absolute DAIs (fractional and total) peak in May–August (possibly related to missing haboob dust) and January–March (potentially linked to missing Harmattan dust, which would be consistent with other models that have too weak NLLJs and underestimate dust emission) [*Fiedler et al.*, 2013]. The overall total absolute DAI peaks through December (2013) to March (2014).

### 3.2 High‐Wind Versus Low‐Wind Situations

**Figure 2:**
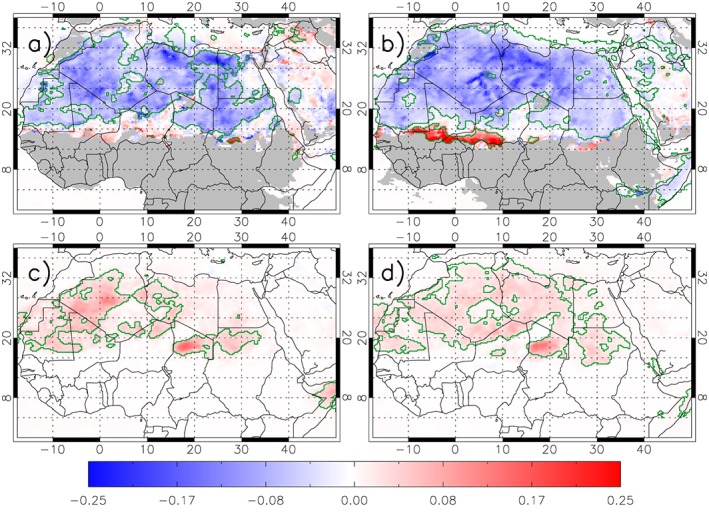
The influence of wind regimes. Model DAI AOD composited under (a and b) high (>7 m/s) and (c and d) low (<7 m/s) model 10 m wind speeds minus the average DAI (see Figures 1c and 1d) during the monsoon season (Figures 2a and 2c) and the nonmonsoon season (Figures 2b and 2d). Green contouring shows significant (95%) differences from the seasonal average DAI using a bootstrapping method (see supporting information).

In order to investigate the dependence of model errors on the meteorology, Figure 2 shows DAI composited for “high” (>7 m/s) and “low” (<7 m/s) 10 m model wind speed regimes (7 m/s is a typical threshold for emission) [*Chomette et al.*, 1999]. Results using 6, 8, or 9 m/s are similar (not shown). Differences in DAI AOD seen in Figures 1 and 2 will arise from both model errors in the land surface and winds.

DAIs are normally negative over the Sahara (i.e., dust is overpredicted, Figure 1). Figures 2a and 2b show that in both seasons, this error increases in magnitude under strong winds (blue); i.e., the model emits more dust than the seasonal average dust overestimation. This is more pronounced in the nonmonsoon season with more significant pixels (green lines—using a “Bootstrapping method”; see supporting information). In contrast, dust is underestimated over the Sahel in strong winds (red), and again, this error is greater than that seen in the mean state. This error in the Sahel is largest in the nonmonsoon season, which could be related to the land surface model struggling to realistically treat the vegetation dieback during the dry season and its effect on roughness. Another factor could be underestimation of NLLJ speed [e.g., *Fiedler et al.*, 2013]. Generally, since dust emission is expected for many of the strong winds analyzed in Figure 2, the similarities between Figures 2a and 2b and Figures 1c and 1d suggest that much of the mean errors seen in Figure 1 arise from errors in emissions.

Under “low winds” (<7 m/s), Figures 2c and 2d show that the model underestimates dust. This is most pronounced over the Sahara during the monsoon season. Together with the overestimation of dust in high winds, this could indicate that the balance between the wind‐based and emission scaling tunings needs to be revisited to reduce the dust emission for high winds, but with a compensating increase emission at lower wind speeds. However, the overprediction in the Sahara decreases with increasing wind (not shown), as models struggle to capture the highest winds [*Cowie et al.*, 2015; *Largeron et al.*, 2015] and the highest AODs often come from haboobs [*Marsham et al.*, 2013; *Allen et al.*, 2013] that are missed by the model. There may also be an impact of a lower resolution DA system attempting to smooth out high AOD features associated with locally high winds.

The underprediction at low winds is consistent with dust raised by processes known to be missing from the model, including cold pools generated by isolated and organized convective storms (haboobs) and subgrid‐scale features such as dust devils. Overprediction of emission at modeled high wind speeds compensates to give a model mean AOD that is close to observed. The role of haboobs is demonstrated in section 3.3. In addition, Figures 1 and 2 show how dust is overestimated from the Bodélé Depression (represented by “B” in Figure 1) on average but underestimated during light winds. This may arise from an insufficient representation of the mountain channeling and NLLJ breakdown in these synoptic conditions [*Todd et al.*, 2008] or perhaps other mesoscale and BL processes. The skill of the UM wind speeds over the Sahara/Sahel is discussed in the supporting information.

### 3.3 Role of Haboobs in Model Errors

**Figure 3:**
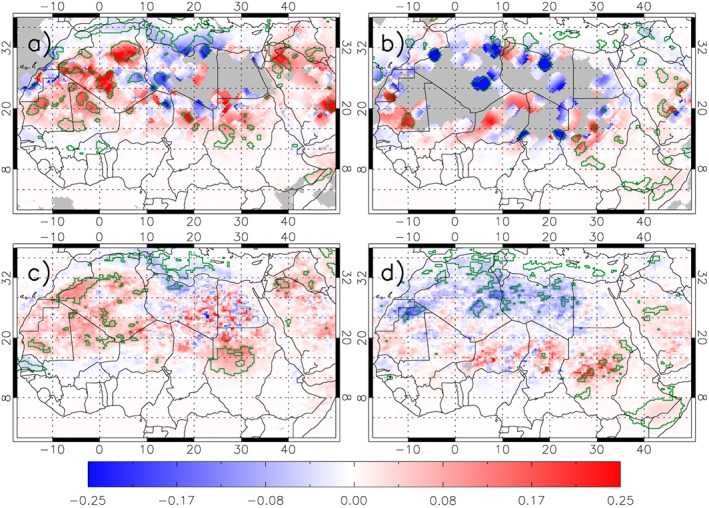
The influence of haboobs on model DAI AOD composited under (a and b) LIS lightning events and (c and d) significant TRMM rainfall minus the average DAI (see Figures 1c and 1d) during the monsoon season (Figures 3a and 3c) and the nonmonsoon season (Figures 3b and 3d).

Haboob winds are known to be largely missing from the model due to the use of parameterized moist convection [*Marsham et al.*, 2011]. Figure 3 shows composites of dust DAI AOD, using two different identifiers of the deep convection that generates haboobs.

Deep convection typically forms over Africa in the late afternoon with large organized systems persisting through the night and decaying the next day [e.g., *Birch et al.*, 2014a, 2014b; *Rickenbach et al.*, 2009]. For major storms the haboobs generated can span scales of several hundreds of kilometers [e.g., *Roberts and Knippertz*, 2014]. Figures 3a and 3b therefore show AOD DAIs composited for a radius of 2° around observed lightning flashes, using the 24 h of LIS flashes observed before the 12 UTC DAI. Similarly, Figures 3c and 3d use the 24 h of TRMM rainfall preceding the 12 UTC DAI, and composites using boxes of 3 by 3 pixels (75 by 75 km) around rainfall events, which are defined as where the 24 h accumulated rain is >25% of the seasonal (monsoon and nonmonsoon) rainfall average for that location. For both TRMM and LIS, changing details of the methods alter small‐scale details of the plots, but not the overall picture. Typically, the TRMM sample size is approximately 20–30% larger than that of LIS over the Sahel and Sahara.

The composites in Figure 3 are noisy. However, using either method, in the monsoon season, there is a significant signal of underprediction of dust where haboobs are expected over the western part of the Sahara all the way north to the southern foothills of the Atlas chain (Figures 3a and 3c) [*Redl et al.*, 2015], with evidence of signals in other regions such as northern Chad and Sudan. This is consistent with past studies showing evidence of summertime haboobs in these regions [*Marsham et al.*, 2013]. The fractional positive DAIs (Figure 1e) peak in May–June, when a dry atmosphere tends to give stronger haboob winds [*Marsham et al.*, 2008a; *Provod et al.*, 2015] representing 10–30% of the model AOD. This potentially represents the haboob emission missed by the model, but some may be from other sources of error, and there may also be compensating errors. In contrast over the Mediterranean, there is a weak and not statistically significant overprediction of dust, which may be linked to wet deposition of suspended dust from midlatitude frontal precipitation.

In the nonmonsoon season (Figures 3b and 3d), when haboobs are less numerous, the signal is rarely significant. There is overprediction over the northern Sahara, where in this season cyclonic or frontal systems generate both rain and lightning and likely remove dust by wet deposition.

## 4 Conclusions

We have developed a novel approach to understand sources of errors in dust forecasts from an NWP system, made possible by assimilation of satellite‐derived AODs. Overall, the assimilation of MODIS AOD into the UK Met Office global model examined here improves comparisons between model dust AOD and AERONET AODs for the year studied. The 1 year analyzed already provides an illustration of the limitations of the model and the possibilities of this new approach. The assimilation shows that the model is underestimating dust emission in the Sahel and overestimating it over the Sahara throughout the year.

The model overpredicts and underpredicts dust under strong (>7 m/s) and light (<7 m/s) model 10 m winds, respectively, relative to the seasonally average errors. This is consistent with mesoscale and BL processes, known to be missing in the model, uplifting dust in reality when modeled grid‐scale winds are too low for uplift. Therefore, we hypothesize that the compensating, greater than observed dust at high model wind speeds results from the tuning of the model.

Proxies for haboobs occurrence have been used to detect the underestimation of dust emission in the model resulting from parameterized convection and the associated inability to represent cold pool dust uplift [*Marsham et al.*, 2011]. DAI composited under the haboob proxies shows model AOD underestimation over the western Sahara, especially in the monsoon season. We conservatively suggest that haboob dust emission, from the increase in DAI colocated with deep convection proxies, represents approximately 10–30% of the full dust loading over the Sahel/southern Sahara during the summer monsoon.

This methodology of using DAIs to examine dust forecast errors is a powerful tool and provides insight in model limitations, which cannot be easily extracted from other sources of information. We propose that DAIs should be used more to characterize model errors and to gauge the success of model developments. Assimilation of AOD from Metasets Spinning Enhanced Visible and Infrared Imager would allow for the assessment of the diurnal cycle dust errors.

## Supporting information



Supporting Information S1
