# Supplementary material for: Identifying errors in dust models from data assimilation
Source: Geophys Res Lett. 2016 Sep 3;43(17):9270–9. doi: 10.1002/2016GL070621 (PMC5082526; doi:10.1002/2016GL070621)
Supplement: Supplementary file 1 — Supporting Information S1 [file GRL-43-9270-s001.pdf]

Supporting Information for "Identifying errors in dust models from data assimilation"

R. J. Pope,[1,2] J. H. Marsham,[1,3] P. Knippertz,[4] M. E. Brooks[5], and A. J. Roberts[1]

[1]Institute for Atmospheric and Climate Science, University of Leeds, Leeds, UK.

[2]National Centre for Earth Observation, University of Leeds, Leeds, UK.

[3]National Centre for Atmospheric Science, University of Leeds, Leeds, UK.

[4]Institute of Meteorology and Climate Research, Karlsruhe Institute of Technology, Karlsruhe, Germany.

[5]Met Office, Exeter, UK

Corresponding author: : R. J. Pope, Institute for Atmospheric and Climate Science, University of Leeds, Leeds, UK. (r.j.pope@leeds.ac.uk)

Introduction

The supporting information outlines the evaluation of the assimilation of MODIS aerosol optical depth (AOD) into the model against surface AERONET sites (S1). The test

for statistical significance, "Bootstrapping", used in the main manuscript is also defined below (S2). The proportion of data assimilation increments (DAI) under high and low wind speed regimes in discussed in S3. S4 and S5 provided information on the evaluation of the model 10-m wind speeds and the model 10-m wind speed increments.

Text S1.

Fig. 1 of the supplementary material shows that assimilation of MODIS AODs improves the correlation between the model and AERONET data at all locations (except Oujda), slightly reducing mean bias everywhere (except Tamanrasset). Other studies, such as [*Liu et al.*, 2011], also show model improvements with assimilation of MODIS AOD. Local pollution may also explain why, although the model tends to over predict AODs in the Sahara around Tamanrasset (Fig. 1 of the main manuscript), it under-predicts at Tamanrasset itself. This could also be a result of orographic circulations at this mountainous site. The model captures the seasonal cycle at all sites (correlations from 0.61 to 0.82 without DA, 0.70 to 0.83 with). Zinder has a more variable seasonal cycle than the other Sahelian sites, perhaps caused by variations in dust transport from the Bodélé Depression upwind.

Text S2.

Green contouring indicates where the data assimilation increment (DAI) aerosol optical depth (AOD) composited under a specific event (e.g. DAI AOD sampled under "high" winds in section 3.3 of the main manuscript) is statistically different from the average state DAI AOD (i.e. Fig. 1c & d of main manuscript). This is based on a "Bootstrapping" method, which tests if an event occurred by chance or not. For each model pixel, the

composite sample size (e.g. the number of “high” wind events) is used to randomly sample the seasonal DAI AOD time series and the average calculated. This is repeated 1000 times to fill a sorted random distribution (low-high) of DAI AOD averages. If the composite DAI AOD value (e.g. high wind speed regime composite value - Figure 3a & b of main manuscript) is outside of the random distributions 5th and 95th percentiles, it is classed as statistically significant and did not occur by chance.

Text S3.

Fig. 2 of the supplementary material shows DAI AOD sampled under low and high wind speeds for the monsoon and non-monsoon seasons weighted by the occurrence of these wind regimes in each season. Therefore, the summation of Supplementary Figure 2a & c and b & d would give the seasonal average DAI AOD seen in Figure 1c & d. In both seasons, the Saharan DAI AODs under each wind regime are generally very similar (-0.2 to -0.05). However, over the Bodélé Depression DAIs are dominated by DAIs from the regime with strong modelled 10-m winds, which could be a result of the model wind errors, the land surface or both. Over the Sahel, in the monsoon season especially, positive DAI AODs (0.01), strongest for light winds, suggest a missing dust source associated with parameterized convection (i.e., missing haboob winds), which is discussed further in section 3.3 of the main manuscript.

Text S4.

Comparisons between the model 10-m wind speeds and HadISD observations (*Dunn et al.* [2012]; www.metoffice.gov.uk/hadobs/hadisd/) at 12 UTC over the Sahara/Sahel show reasonable agreement with biases of 0-3 m/s and correlations of 0.5-0.8. From the Cascade UM simulations [*Marsham et al.*, 2011; *Heinold et al.*, 2013] the dust uplift potential and emission in similar models over West Africa has a main peak in the morning, which is strongest at 0700-0900 LT, but misses a second peak in the afternoon (associated with convective cold pools), which is present when convection is explicit.

Text S5.

Fig. 3 of the supplementary material shows the model 10-m wind speed increments for a) the monsoon season and b) the non-monsoon season. Due to the lack of observations over the Sahara, there is little change in the model wind speeds from the assimilation. Therefore, despite errors in the model winds [*Largeron et al.*, 2015; *Cowie et al.*, 2015], the wind speed increments are small.

References

Cowie, S. M., J. H. Marsham, and P. Knippertz (2015), The importance of rare, high-wind events for dust uplift in northern africa, *Geophysical Research Letters*, *42*(19), 8208–8215.

Dunn, R. J. H., K. M. Willet, P. W. Thorne, E. V. Wooley, I. Durre, A. Dai, D. E. Parker and R. S. Vose (2012), HadISD: a quality-controlled global synoptic report database for selected variables at long-term stations from 19732011, *Climate of the Past*, *8*, 1649–1679.

Heinold, B., P. Knippertz, J. H. Marsham, S. Fiedler, N. S. Dixon, K. Schepanski, B. Laurent, and I. Tegen (2013), The role of deep convection and nocturnal low-level jets for dust emission in summertime west africa: Estimates from convection-permitting simulations, *Journal of Geophysical Research: Atmospheres*, *118*(10), 4385–4400.

Largeron Y., F. Guichard, D. Bouniol, F. Couvreux. L. Kergoat and B. Marticorena (2015), Can we use surface wind fields from meteorological reanalyses for Sahelian dust emission simulations?, *Geophys. Res. Lett.*, *42*, 2490–2499.

Liu Z., Q. Liu, H.-C. Lin, C. S. Schwartz, Y.-H. Lee, and T. Wang (2011), Three-dimensional variational assimilation of MODIS aerosol optical depth: Implementation and application to a dust storm over East Asia, *J. Geophys. Res.*, *116*(D23206).

Marsham, J. H., P. Knippertz, N. S. Dixon, D. J. Parker, and G. M. S. Lister (2011), The importance of the representation of deep convection for modeled dust-generating winds over west africa during summer, *Geophysical Research Letters*, *38*(16).

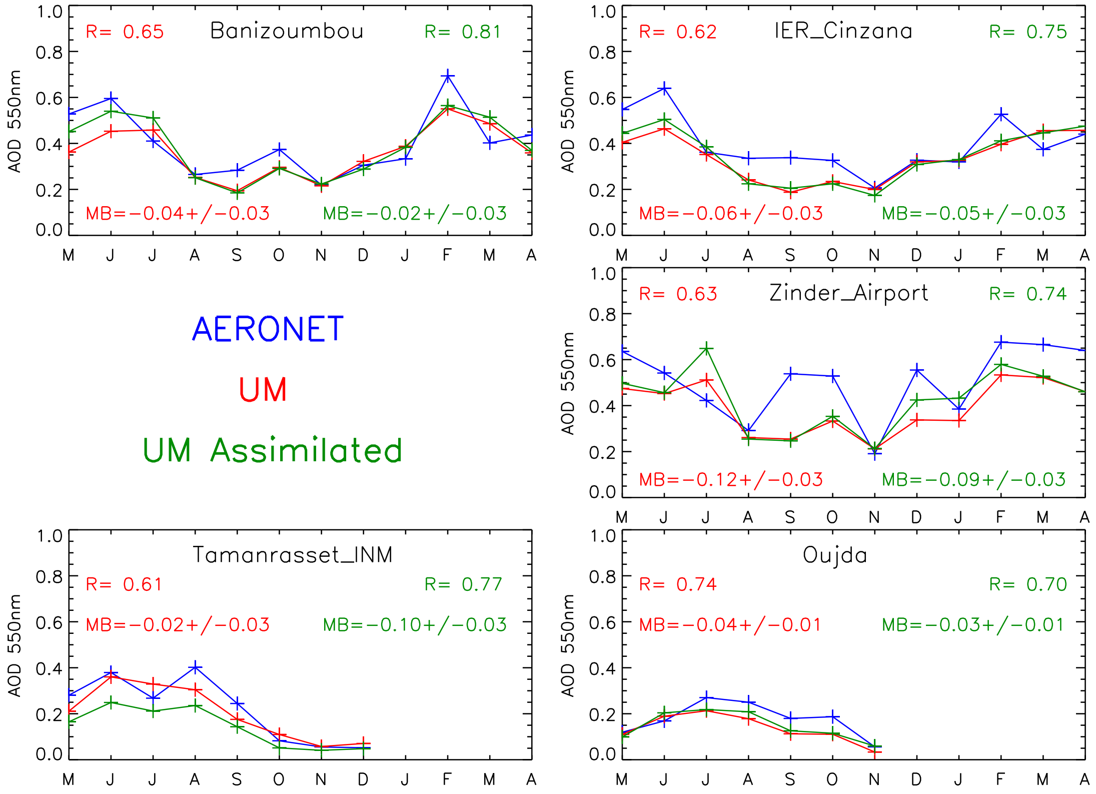

Figure 1. Model evaluation: Seasonal cycles of modelled dust AOD with (green) and without (red) assimilation of MODIS and co-located observations from AERONET (blue; see white and red symbols for locations in Fig. 1 of the main manuscript). Square = Tamanrasset, star = Cinzana, triangle = Oujda, X = Zinder Airport and + = Banizoumbou. B represents the Bodélé Depression. The mean bias (MB) error bars are the standard error in the AERONET data with the autocorrelation taken into account. R is correlation.

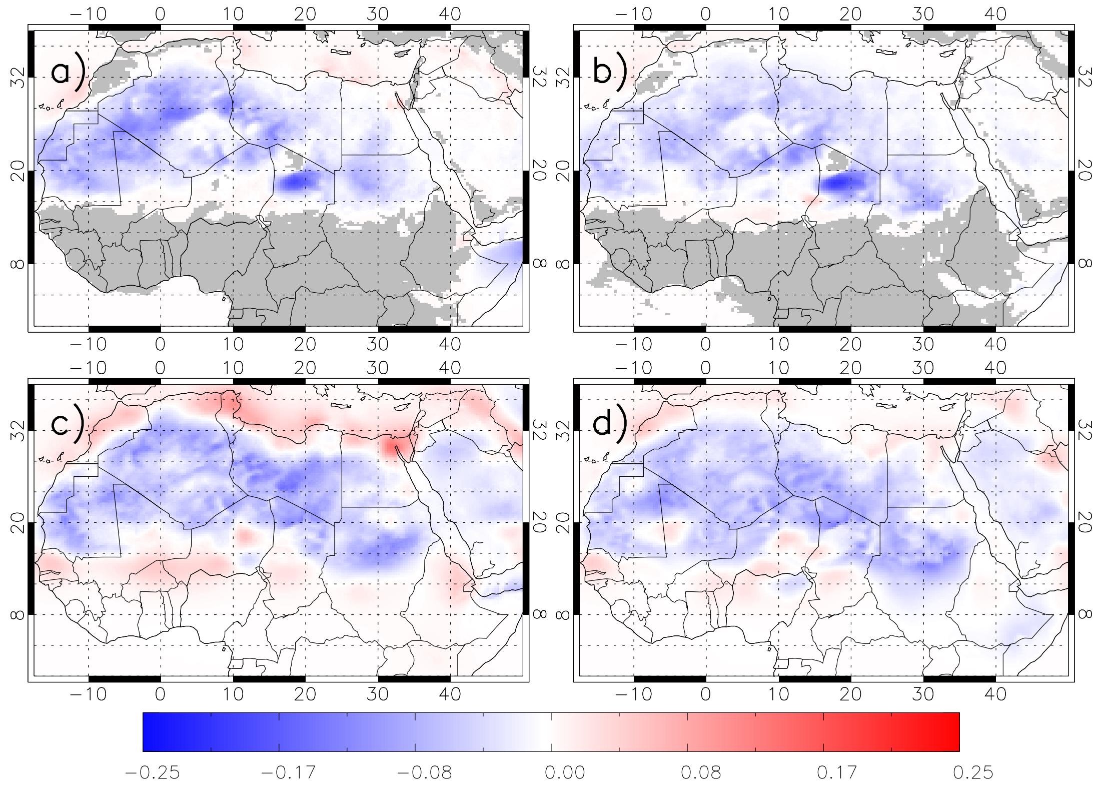

Figure 2. Contribution to the mean model DAI AOD under high (>7 m/s, top) and low (<7 m/s, bottom) model 10-m wind speeds during the monsoon season (left) and the non-monsoon season (right).

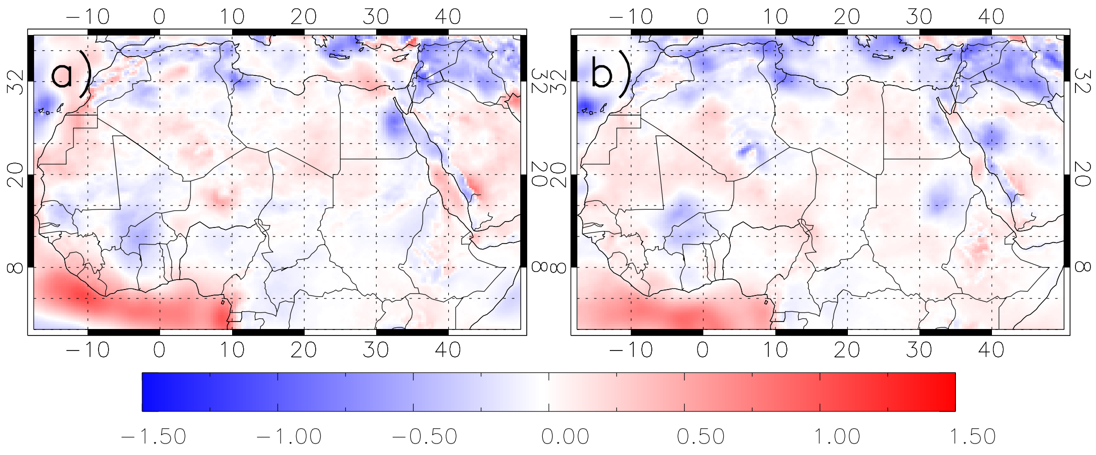

Figure 3. Model 10-m wind speed increments (m/s, 12 UTC), for a) the monsoon season and b) the non-monsoon season.
